# Image Processing Technique and Hidden Markov Model for an Elderly Care Monitoring System

**DOI:** 10.3390/jimaging6060049

**Published:** 2020-06-13

**Authors:** Swe Nwe Nwe Htun, Thi Thi Zin, Pyke Tin

**Affiliations:** 1 Interdisciplinary Graduate School of Agriculture and Engineering, University of Miyazaki, Miyazaki 889-2192, Japan; 2 Graduate School of Engineering, University of Miyazaki, Miyazaki 889-2192, Japan; thithi@cc.miyazaki-u.ac.jp; 3 International Relation Center, University of Miyazaki, Miyazaki 889-2192, Japan; pyketin11@gmail.com

**Keywords:** normal and abnormal states, fall detection, Mixture of Gaussians, graph cut, virtual grounding point, Hidden Markov Model

## Abstract

Advances in image processing technologies have provided more precise views in medical and health care management systems. Among many other topics, this paper focuses on several aspects of video-based monitoring systems for elderly people living independently. Major concerns are patients with chronic diseases and adults with a decline in physical fitness, as well as falling among elderly people, which is a source of life-threatening injuries and a leading cause of death. Therefore, in this paper, we propose a video-vision-based monitoring system using image processing technology and a Hidden Markov Model for differentiating falls from normal states for people. Specifically, the proposed system is composed of four modules: (1) object detection; (2) feature extraction; (3) analysis for differentiating normal states from falls; and (4) a decision-making process using a Hidden Markov Model for sequential states of abnormal and normal. In the object detection module, background and foreground segmentation is performed by applying the Mixture of Gaussians model, and graph cut is applied for foreground refinement. In the feature extraction module, the postures and positions of detected objects are estimated by applying the hybrid features of the virtual grounding point, inclusive of its related area and the aspect ratio of the object. In the analysis module, for differentiating normal, abnormal, or falling states, statistical computations called the moving average and modified difference are conducted, both of which are employed to estimate the points and periods of falls. Then, the local maximum or local minimum and the half width value are determined in the observed modified difference to more precisely estimate the period of a falling state. Finally, the decision-making process is conducted by developing a Hidden Markov Model. The experimental results used the Le2i fall detection dataset, and showed that our proposed system is robust and reliable and has a high detection rate.

## 1. Introduction

The risk of falling is a huge issue, not only for older adults in aging societies, but also for younger adults with drug and alcohol problems and for patients with chronic diseases. Falling has serious health consequences and is a leading cause of death. According to a survey by the World Health Organization [1], falling occurs most frequently in the 28–35% of individuals between the ages of 65 and 70 and in the 42% who are over 70 years old. Falling is particularly dangerous for persons who live alone in an indoor environment, because much time can pass before they receive assistance. In this situation, many countries are adopting policies to increase the life expectancy, by providing extra care to people living independently.

For this reason, much research has focused on developing a robust fall-detection process in smart home systems using their respective specialized technologies. Progress in developing such intelligent technologies holds the promise of improving the quality of life for the aged and infirm. Popular fall detection and prediction systems have emerged based on wearable monitoring devices, ambient devices, vision-based devices, and portable devices. The most commonly used wearable devices feature accelerometers and gyroscopes embedded in belts, watches, or pendants that are reasonably comfortable to wear. However, some of these devices have disadvantages that limit their usability, such as excessive power consumption. Some of these devices produce false alarms when triggered by normal body movements, and some require the manual activation of an alarm after a fall. In addition, the elderly often forgets to wear the devices. However, wearable devices based on machine learning and intelligent systems are effective for monitoring people in both indoor and outdoor environments. Various interesting approaches for fall detection systems using wearable devices are discussed in [2,3,4,5], in which accelerometers and gyroscopes are employed to collect data on the emotional state and body movements of subjects.

The processing of data collected using ambient devices provides information without demanding user intervention. Commercially available devices using advanced technologies include presence sensors, motion sensors, and vibration sensors, which can be embedded in an indoor environment, such as on furniture. Presence sensors can detect the tiniest movements of subjects, such as the movement of a finger, with a high resolution and precision. They can easily be set on high ceilings, as well as the floor, to detect the smallest movement. Compared to presence sensors, motion sensors are useful in perceiving the arm movements involved in walking in detection zones. Such zones are selected as high-traffic areas for detecting moving objects in busy indoor or outdoor areas. Vibration sensors are useful for detecting falling events, and can distinguish the activities through vibrations. Vibration sensors installed on floors or beds acquire vibration data that can be analyzed by determining the fall detection ratio [6]. Although such sensors do not disturb the people involved, they can generate false alarms.

In recent years, the development of smart phone technologies that are incorporated with accelerometers has provided some very interesting approaches for healthcare monitoring systems, including fall detection. Mobile phone-based fall detection algorithms have been presented in [7,8,9,10]. However, these devices are not useful if the monitored subject does not always have them in their hand.

For these reasons, video monitoring systems based on computer vision and machine learning are potentially more beneficial and reliable for fall detection. Vision-based devices utilizing cameras have some of the same limitations as ambient devices, such as the fact that the devices must be installed in several places to provide full coverage of the required areas over a long period of time. However, video surveillance systems can effectively predict specific human activities, such as walking, sitting down, going to bed, and getting up from bed [11,12], as well as detecting fall events [13]. Moreover, a large amount of visual information is captured in the video record of falling events. The various definitions of falling events, as well as the reasons and circumstances behind such events and other abnormal occurrences in real-world environments, should be visually analyzed in monitoring systems. The vision-based detection of abnormal or fall events could become an important tool in elderly care systems.

In developing better vision-based video monitoring systems, abnormal event detection holds great promise, but faces many challenges in real-world environments. In developing applications for this purpose, establishing a fall detection system presents difficulties because the dynamic conditions involved in falls are not well-understood. As an important focus, researchers must investigate the universal features common to all falling events. Therefore, we must differentiate falls from normal activities in a comprehensive way that reflects the scene of falling scenarios. Any system developed must be robust in the face of changing postures and positions, such as a loss of balance, and abrupt changes in direction. These conditions must be considered in reliably assessing abnormal events. However, research trends and current best practices indicate that the prospects are great for using vision-based monitoring to improve the quality of health care.

Therefore, we propose a vision-based system for fall detection by differentiating abnormal behavior or falls from normal states. This system includes simple and uncomplicated feature extraction or effective statistical analyses. Key to detecting abnormal or falling events is the recognition that they involve a loss of balance. The three research objectives of this proposed system are as follows:

- Develop a monitoring system that provides a visual understanding of a person’s situation and can judge whether the state is abnormal or normal based on video data acquired using a simple and affordable RGB camera;
- Develop an individualized and modified statistical analysis on each of the extracted features, providing trustworthy information, not only on the definite moment of a fall, but also on the period of a fall;
- Develop an efficient way of using a Hidden Markov Model (*HMM*) for the detailed detection of sequential abnormal and normal states for the person being monitored.

Our proposed system intends to establish a long-term monitoring system for facilitating independent living. Major steps in the system include (1) feature extractions in estimating positions and postures of the person by utilizing the virtual grounding point (*VGP*) concept, and related visual features inspired by our previous research [13]; (2) modified statistical analysis to estimate the time interval or period for falls and normal states through extensive feature observation; and (3) the establishment of a Hidden Markov Model (*HMM*) to detect the sequential normal and fall states of the person. The rest of the paper is organized as follows: Section 2 presents related works; Section 3 presents theoretical analysis and methodologies of the proposed system; Section 4 presents and evaluates experimental results, comparing the robustness and limitations of the proposed systems with existing algorithms; and finally, Section 5 presents the conclusions of this work.

## 2. Related Works

Related state-of-the-art fall detection systems will be discussed in this section. In order to effectively define the postures assumed by a human object, it is very important to perform feature extraction or selection, analyze the selected features, and set detection rules in a vision-based video monitoring system, especially for monitoring in health care. The most common feature extraction methods used in fall detection systems involve the human shape and motion-history images. The fall detection system presented in [14] is a method based on motion history images (*MHI*) and changes in the human shape. In this system, information on the history of motion occurring over a fixed interval can be obtained from *MHI*. Then, the human shape is obtained by constructing a blob using an approximate ellipse. Finally, fall detection is achieved by considering three factors: motion quantification, analysis of the human shape, and the lack of motion after a fall. Motion quantification allows sudden motion to be detected when a person falls. The approximated ellipse constructed on the object can provide information about changes in the human shape, more precisely, changes in orientation. The final analysis provides a moving ellipse, which indicates whether the person is moving after a fall. The decision confirming a fall is made when the ellipse stops moving for five seconds after a fall. The video sequences are captured using wall-mounted cameras to cover wide areas, and the results are presented in 2D motion and shape information. Extended work on the system presented in [15] involves both 2D and 3D information for fall detection, as the researchers intended to recover localization information on the person relative to the ground. This extended process of feature extraction involves computing the 3D head trajectories of a person, and a fall is detected if the velocity of the head exceeds a certain value and the position of the head is too close to the floor or the ground.

Similarly, a process of detecting unnatural falls has been developed [16], which includes background subtraction using the frame difference method, feature extraction using *MHI*, and a change in human shape and classification using a support vector machine (*SVM*). Compared with the previous approach [15], the system involves constructing three specific features on the human shape, namely, the orientation of the approximated ellipse, its aspect ratio, and silhouette detection below a threshold line. The aspect ratio of the ellipse describes changes in major and minor axes, and differentiates a fall from normal activities. After a fall, the previously moving object lies on the ground. For this reason, the threshold line is set by considering a suitable height from the ground. After that, fall and non-fall objects can be differentiated according to the height of the silhouette object. Finally, classifications are performed using the support vector machine (*SVM*), k-nearest neighbor (*KNN*) classifier, Stochastic Gradient Descent (*SGD*), Decision Tree (*DT*), and Gradient Boosting (*GB*). The *DT* algorithm consistently outperforms the rest, with a high detection rate, as confirmed using the Le2i fall detection dataset.

Another fall detection system for elderly care is proposed in [17]. This system firstly conducts background subtraction to segment out the silhouette moving object, and then tracks the object to determine the trajectory. Secondly, a timed motion history image (*tMHI*) is constructed to detect high velocities. Then, the motion is quantified to acquire pixel values for the *tMHI*, which are divided by the number of pixels in the detected silhouette object. Similar to the approach presented in [16], this system provides a useful definition of the human body posture using the following combined features as the input: the ratio, orientation, and major and minor semi-axes of the fitted ellipse. In this system, both *MHI* and a projection histogram are applied to confirm that a falling event has occurred. In addition, the position of the head can be tracked in sequential frames in order to obtain useful information, since the trajectory of the head is visible most of the time. Finally, a multilayer perceptron (*MLP*) neural network is applied on the extracted features to classify falls and non-falls, with an accuracy of 99.24%, and a precision of 99.60%, as confirmed using the UR fall detection dataset. In addition, an extensive automated human fall-recognition process is proposed in [18] to support independent living for the elderly in indoor environments using the Le2i fall-detection dataset. This system is based on motion, orientation, and histogram features, and achieves an overall accuracy of 99.82%. The approaches in [14,15,16,17,18] discussed above focus on distinguishing falls from normal activities.

However, any monitoring system should take into account consecutive daily activities in a real-world environment. Such activities include walking, standing, and sitting, as well as transitioning between these activities. In this regard, our previous work [19] proposed human action analysis based on motion history and the orientation of the human shape. As its main purpose, this system takes into consideration a prediction of the degree of mobility for the elderly in daily activities, such as getting into and out of bed. These activities include the following consecutive actions: sitting, transitioning from sitting to lying, lying, transitioning from lying to sitting, transitioning from sitting to standing, and walking. We firstly conducted background subtraction [20,21] for the proper separation of foreground and background objects. Among the features used in this system are *tMHI* and the orientation of the approximated ellipse. However, the construction of one ellipse is not enough for detecting the human object region. As a key part of our design, two approximated ellipses are constructed using horizontal and vertical histogram values. The vertical histogram is employed for the whole body region, and the horizontal histogram is employed for the upper body region. Then, motion quantification is used to analyze these human activities. In considering detailed sequential actions, multiple threshold values are observed, which depend on the shape orientation and the coefficient of motion. Rather than using fall scenes, the experiments were conducted using the normal scenes in the 14 videos of the Le2i dataset. After that, we extended our system [22] to analyze not only normal activities, but also falls. In this system, the virtual grounding point (*VGP*) is introduced for feature extraction, and analyses are performed by the combined features of *tMHI* and *VGP*. The overall accuracy of the proposed system for the detection of falling events was 82.18%, as experimentally confirmed using 15 videos from the Le2i fall detection dataset. To improve the accuracy of detecting falls in a given video sequence, we extended our research [13] by incorporating feature selection using the *VGP* concept with its related features and statistical analysis for estimating the falling period, as well as two classification methods, namely the support vector machine (*SVM*) and period detection (*PD*). A comparison with existing approaches using the Le2i dataset showed that our *SVM* approach outperforms the rest, with a precision of 93%, a recall of 100%, and an accuracy of 100%. However, our previous system exclusively concentrated on differentiating falling events from normal events; in other words, classifying the videos which contain abnormal or normal events. A long-term monitoring system for home care requires the effective detection of sequential normal and abnormal states.

Another vision-based fall detection system using a convolutional neural network has been developed by Adrian [23]. This system was designed to work on human motion, avoiding any dependence on the appearance of an image. An optical flow image generator is utilized to efficiently represent human motion. A set of optical flow images are stacked and then set as inputs in a convolutional neural network (*CNN*) that can learn longer time-related features. A fully connected neural network (*FCNN*) receives these features as inputs, and produces fall and non-fall outputs. The best overall accuracy for this system is 97%, as confirmed using the Le2i fall-detection dataset. Moreover, the approach used in [24] proposes fall detection based on body keypoints and sequence-to-sequence architecture. In this system, a skeleton framework is modeled to receive a sequence of observed frames. Then, the coordinates of keypoints of the object are extracted from observed frames. The bounding boxes of the detected object are given to the tracking algorithm for clustering body keypoints belonging to the same person in different video sequences. A keypoint-vectorization method is exploited to extract salient features from associated coordinates. Next, the pose prediction phase is conducted, predicting the vectors of future keypoints for the person. Finally, falls are classified using the Le2i dataset, achieving an accuracy of 97%, a precision of 90.8%, a recall of 98.3%, and an F1-score of 0.944.

A related approach is presented in [25] in an automatic fall-detection system with an RGB-D camera using a Hidden Markov Model (*HMM*). Background subtraction is firstly performed by averaging the depth map to learn the background. The Kinect optical parameters for factory use are employed to obtain a real-world coordinate system, and an Open Natural Interaction (*OpenNI*) is used for a Kinect-to-real-world transformation. After that, the center of mass of the person is extracted to calculate the vertical speed from that point. The standard deviation for all points belonging to a person are then calculated. After that, these three features are used as inputs to calculate the probability of *HMM*. Finally, the forward-backward and Viterbi algorithms are applied to classify the states of normal activities and falls. The experiments were conducted on young and healthy subjects, and occlusions were not included. In future research, this system will be tested using real-life unhealthy subjects with occlusions. In addition, other fall detection systems for shop floor applications [26] were modeled using an *HMM* based on the vertical velocity, area variance, and height of a person, using cameras positioned to provide a top view. This incident detection method focuses on two things: detecting people in restricted areas and detecting falls. Potential fall events are analyzed based on specific features and on circumstances such as whether the person can get up or not. Analysis is also based on an allocation of status according to the location, whether the event occurs in an area that is restricted to all personnel, where work is ongoing, or where maintenance is being performed. Such considerations are valuable in identifying health and safety issues. In future work, this system will be improved by incorporating more incidents, such as collisions.

Vision-based monitoring systems for detecting falls or abnormal events can be powerful tools in various applications. These new technologies have great potential as intelligent monitoring systems. Therefore, we propose a detection system for indoor environments that include normal activities; one that is based on the consecutive states of abnormal or fall events using image processing techniques and a Hidden Markov Model. This detection system will be ideal for elderly care monitoring systems.

## 3. Proposed Architecture of the System

**Figure 1:**
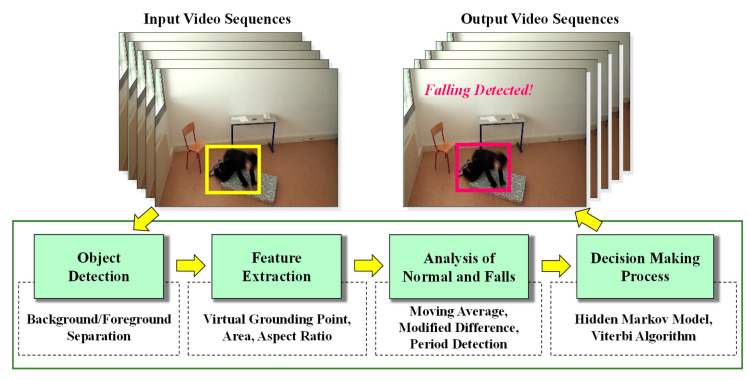
The major work flow of our proposed system.

The proposed monitoring system provides a way to detect normal, abnormal, and falling states for persons being monitored, such as seniors with a limited mobility, patients with chronic diseases, and those recovering from surgical procedures. The proposed system is composed of the following four main modules: (1) object detection; (2) feature extraction using the virtual grounding point (*VGP*) concept; (3) analysis of normal, abnormal, or falling events; and (4) establishment of decision-making rules utilizing a Hidden Markov Model (*HMM*). The major work flow for our proposed system is illustrated in Figure 1, and specialized theoretical analyses for each module are described in the following sections.

### 3.1. Object Detection

This module features an object detection procedure similar to that used in our previous work [13], and is briefly described here. We select a specific Mixture of Gaussians (*MoG*) distribution [20] for modeling the foreground, which is updated frame by frame. We also use specific low-rank subspace learning for modeling the background for each successive frame in the video sequences. After that, an expectation and maximization (*EM*) algorithm is used for updating the foreground and background parameters of each new frame. However, the quality of the foregrounds is unsatisfactory because ghost effects are included in the resultant foregrounds. In real-life video sequences, as well as in simulated video sequences, much redundant data occurs, such as when a person moves very slowly or stays in place for a long period of time. In such situations, most systems have trouble recognizing a person as the foreground. To overcome this problem, a graph cut algorithm is applied to refine the foreground [21]. A set of the resulting foreground and background frames is given as inputs for the video sequences. Then, we seek binary labels regarded as the foreground, which is set to 1, and the background, which is set to 0. These foreground and background labels are calculated by constructing a graph structure, *G* = (*v_vertex_*, *ε*), where *v_vertex_* is the set of vertices or pixels and *ε* is the set of edges linking the closest four-connected pixels [21]. The maximum-flow and minimum-cut method is used to find the vertex label with a minimum energy function. Most traditional graph cut algorithms work on manual drawings of the scribbles and region of interest for the targeted object in every frame. In applying graph cut theory for refining the foreground, we obtain the associated *MoG* foreground mask in every 100th frame, instead of repeatedly re-drawing the scribbles and region of interest. The combination of these two effective methods optimizes the solution for object detection.

### 3.2. Feature Extraction

In this module, feature extraction is performed by applying the virtual grounding point (*VGP*), as well as the area and aspect ratio of the object [13]. The computation strategies for feature extraction are simple and effective. Using these strategies, we adapted our previously proposed method of feature extraction [13] in this extension of our work. Here, we briefly describe our previous research using the *VGP* concept. A virtual grounding point is a point which is constructed for exploring the position and posture of humans. Specialized technical details are described as follows.

Let *p* be the position at time *t* on the detected silhouette object. Then, the centroid of object *C*(*t*) is extracted as defined in Equation (1):

(1) $$C\left(t\right)=\left(x_{c}\left(t\right),y_{c}\left(t\right)\right),$$

where *t* represents *p*(*t*) = (*x*(*t*),*y*(*t*)), and *x_c_* and *y_c_* denote that $x_{c}=\sum_{i=1}^{N}\left(x_{i}/N\right)$ and $y_{c}=\sum_{i=1}^{N}\left(y_{i}/N\right)$ respectively.

Then, a vertical line from the top-most to the bottom-most row corresponding to the *x* axis of *C*(*t*) is constructed on the detected object. Similarly, a horizontal line from the left to the right column corresponding to the *y* axis of *C*(*t*) is constructed. Finally, a central point where the vertical line along the *x* axis crosses the horizontal line along the *y* axis is marked as a *VGP*, which can be defined as in Equation (2):

(2) $$VGP\left(t\right)=\left(x_{VGP}\left(t\right),y_{VGP}\left(t\right)\right),$$

where the *VGP* is a virtual grounding point at time *t*.

Then, the point distance (*d*) between the centroid *C*(*t*) and its corresponding virtual grounding point *VGP*(*t*) is formulated as shown in Equation (3):

(3) $$d\left(t\right)=\left|y_{VGP}\left(t\right)-y_{c}\left(t\right)\right|,$$

where *d*(*t*) can be denoted as the point distance between centroid *C*(*t*) and its corresponding virtual grounding point *VGP*(*t*) on the detected object at time *t* to extract the features for the position and posture of the person.

After that, the area of the object shape (*a*) can be derived to obtain knowledge for the shape regularity of the moving object, as described in Equation (4):

(4) $$a\left(t\right)=\left(\sum_{i=1}^{N}\sum_{j=1}^{N}I\left(i,j\right),t\right),$$

where *a*(*t*) is the measurement of the area indicating the relative size of the object obtained by summing all the pixels in the object at time *t*.

**Figure 2:**
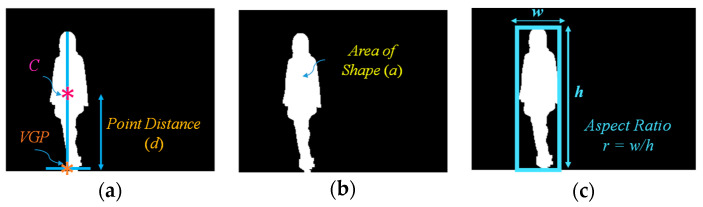
Feature extraction: (**a**) Point distance (*d*); (**b**) area of the object (*a*); (**c**) aspect ratio of the object (*r*).

Finally, the aspect ratio (*r*) with respect to the object is simply calculated to estimate the human posture as shown in Equation (5):

(5) r(t)=w(t)/h(t),

where *r*(*t*) is the aspect ratio of the object found by dividing the value of the width (*w*) by that of the height (*h*) for the object at time *t*. The concepts for deriving the individualized features are illustrated in Figure 2.

### 3.3. Analysis of Abnormal and Normal Events

For detecting abnormal or falling events, we first computed the moving average (*MA*) [13] using features extracted from sequential data. In our previous research [13], we used two features (point distance (*d*) and aspect ratio (*r*)) for analyzing the data points of the moving object. In this extension of our work, statistical analysis of the area of the object shape on the observable data series is used with one additional feature. As a result, our proposed formulation uses the following three features: (1) the point distance (*d*) between the centroid and the corresponding virtual grounding point; (2) the area of the object shape (*a*); and (3) the aspect ratio of the object (*r*), as shown in Equation (6):

(6) $$MA\left(t,F,N\right)=\frac{1}{2N+1}\sum_{f=t-N}^{f=t+N}F\left(t\right),F\left(t\right)=d\left(t\right),a\left(t\right),r\left(t\right),$$

where *MA*(*t*,*F*,*N*) is an output value for the average period in *N* at time *t*, *N* represents the number of periods, and *F*(*t*) is the calculation on the three extracted features: the point distance (*d*), the area of the object shape (*a*), and the aspect ratio (*r*) at time *t*. The optimized threshold (*Th*) value is considered in detecting abnormal or falling events. Here, we set the threshold value of *N* at *Th*(*MA*(*t*,*d*,*Th*)), *Th*(*MA*(*t*,*a*,*Th*)), and *Th*(*MA*(*t*,*r*,*Th*)), where *Th* = 51, which depends on the video frame rate. The process for selecting the optimal threshold is similar to that used in our observations in previous research [13]. Calculating the moving average for the sequential data approximates irregular and falling events by observing the crossing point on primary and average feature points.

After that, we define the difference calculation, specifically the modified difference (*MD*), for more precisely estimating abnormal and falling states on the crossing point of the moving average (*MA*), as shown in Equation (7):

(7) $$MD\left(t,F,N_{0},N_{1}\right)=MA\left(t,F\left(t+N_{0}+N_{1}\right),N_{1}\right)-MA\left(t,F\left(t-N_{0}-N_{1}\right),N_{1}\right),\;F\left(t\right)=d\left(t\right),a\left(t\right),r\left(t\right),$$

where *MD*(*t*,*F*,*N*_0_,*N*_1_) denotes the modified difference for the three selected features (*d*, *a*, and *r*) at time *t* and here, the selected optimal value of the threshold for *N*_0_ = 0 and *N*_1_ = 51.

**Figure 3:**
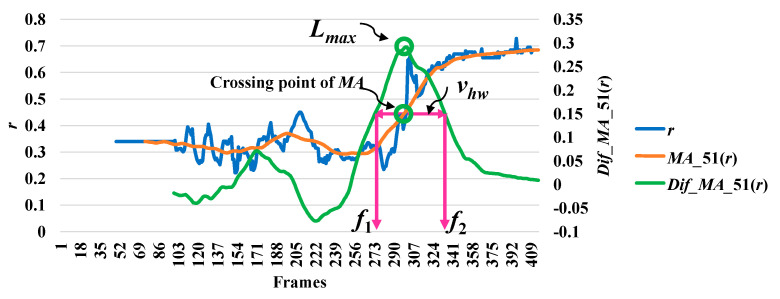
Analysis for a high possibility of abnormal (falling) points based on the aspect ratio (*r*).

**Figure 4:**
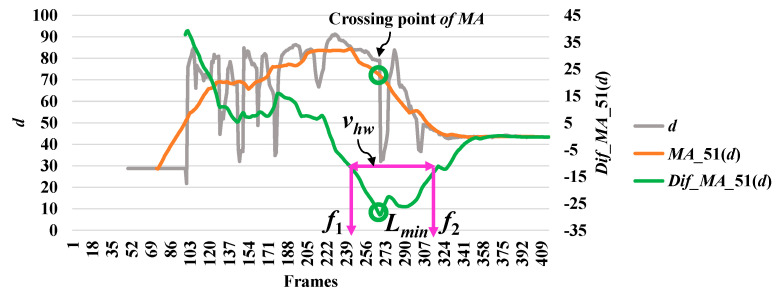
Analysis for a high possibility of abnormal (falling) points based on the point distance (*d*).

In analyzing a series of data based on the point distance (*d*), the modified difference (*MD*) reached a minimum point, which indicates a high possibility of a fall. The modified difference observed for the aspect ratio (*r*) of the person indicates a high possibility of a fall with a maximum point. Here, we say that the highest point is the local maximum (*l_max_*) and the lowest point is the local minimum (*l_min_*). The concepts of discovering a point which is important in referring an abnormal state or a fall by utilizing *r* and *d* are illustrated in Figure 3 and Figure 4, respectively.

**Figure 5:**
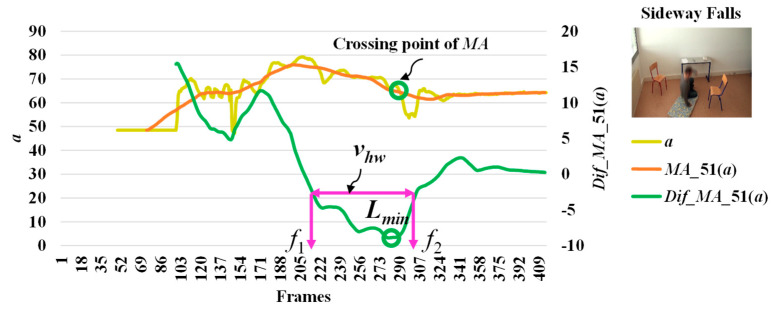
Analysis for a high possibility of abnormal (falling) points based on the area of the object shape (*a*) by falling sideways.

**Figure 6:**
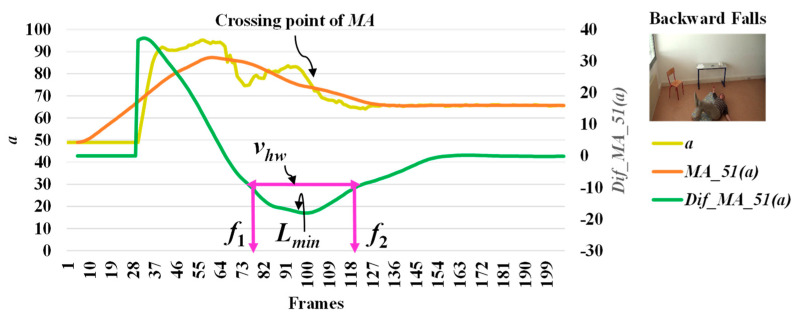
Analysis for a high possibility of abnormal (falling) points based on the area of the object shape (*a*) by falling backwards.

**Figure 7:**
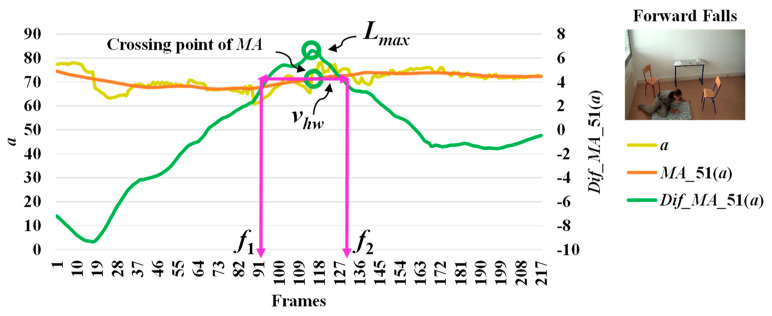
Analysis for a high possibility of abnormal (falling) points based on the area of the object shape (*a*) by falling forwards.

When we observe stationary points for the modified difference on the area of the object shape (*a*), we make an interesting discovery that both *l_max_* and *l_min_* occur with respect to the falling posture and direction of the object. The observations for *l_max_* and *l_min_* which correspond to the different falling postures and positions (sideways falls, forward falls, and backward falls) are described in Figure 5, Figure 6 and Figure 7, respectively. This observation and analysis of the area of the object shape is part of the extension of our previous work [13], and can be properly used in further observations to develop decision-making rules.

Then, we analyze the interval or period of the abnormal event by computing the half width value (*v_hw_*) [13] on the observed curve of modified difference (*MD*), as formulated in Equation (8). The half width value (*v_hw_*) more accurately estimates the period of abnormal and normal events. At the halfway point on the largest curve obtained from the computation of *MD*, the starting point (*f*_1_) and ending point (*f*_2_) are set to represent irregular fall events. After that, the periods of events are observed by calculating the distance of the two points *f*_1_ and *f*_2_.

(8) $$v_{hw}=\left|f_{1}-f_{2}\right|,$$

where *v_hw_* is the estimated period for falling assumed by the half of the curve including *l_max_* or *l_min_*, and *f*_1_ and *f*_2_ represent the starting and ending points of a fall, respectively. Illustrations for developing a better understanding of *v_hw_* are embedded in Figure 3, Figure 4, Figure 5, Figure 6 and Figure 7.

In Figure 3, the analysis for a high possibility of abnormal (falling) points is illustrated based on the aspect ratio (*r*) with respect to the crossing point of the moving average (*MA*), local maximum (*l_max_*), and half width value (*v_hw_*) obtained from the distance between *f*_1_ and *f*_2_. Figure 4 describes a high possibility of abnormal points based on the point distance (*d*) with respect to the crossing point of the moving average (*MA*), local minimum (*l_min_*), and half width value (*v_hw_*) obtained from the distance between *f*_1_ and *f*_2_. By observing the area of the object shape (*a*), we noticed that the local minimum (*l_min_*) occurs in the posture involved in falling sideways and backwards, as demonstrated in Figure 5 and Figure 6, respectively. In Figure 7, the local maximum (*l_max_*) occurs for the posture involved in falling forward when analyzing a high possibility of falling points based on the area of the object shape.

Finally, we consider the threshold values of the time intervals for abnormal and normal events based on the half width value (*v_hw_*) in a given video sequence. Let *A* = {*a*_1_,*a*_2_,…,*a_k_*} represent the videos that include the abnormal events, and *N* = {*n*_1_,*n*_2_,…,n*_k_*} represent the videos composed of the normal events. After that, we calculate

(9) $$\alpha_{1}=\min\left(v_{hw}\right)\in A,\;\alpha_{2}=\max\left(v_{hw}\right)\in N,.$$

Then, the mid value or falling period detection (*PD*) is calculated for three features, namely, the point distance (*d*) between the centroid and its corresponding virtual grounding point of the object, the area of the object shape (*a*) as an extensive feature observation, and the aspect ratio (*r*), which are formulated as shown in Equations (10)–(12), respectively.

(10) $$PD\left(d\right)=\frac{\left(\alpha_{1}\left(d\right)+\alpha_{2}\left(d\right)\right)}{2},\left\{ \begin{array}{ll} l_{1} & \mathrm{if}\;v_{hw}\geq PD\left(d\right)\\ l_{2} & \mathrm{otherwise} \end{array}\right.,$$

(11) $$PD\left(a\right)=\frac{\left(\alpha_{1}\left(a\right)+\alpha_{2}\left(a\right)\right)}{2},\left\{ \begin{array}{ll} l_{1} & \mathrm{if}\;v_{hw}\geq PD\left(a\right)\\ l_{2} & \mathrm{otherwise} \end{array}\right.,$$

(12) $$PD\left(r\right)=\frac{\left(\alpha_{1}\left(r\right)+\alpha_{2}\left(r\right)\right)}{2},\left\{ \begin{array}{ll} l_{1} & \mathrm{if}\;v_{hw}\geq PD\left(r\right)\\ l_{2} & \mathrm{otherwise} \end{array}\right.,$$

where *α*_1_ and *α*_2_ represent the minimum and maximum threshold values used to estimate the period of abnormal and normal events, respectively; *l*_1_ represents the class label for the given video that includes an abnormal event; and *l*_2_ represents the class label which indicates that the given video does not include an abnormal event.

### 3.4. Decision-Making Rules

As the main contributions of this research, we developed considerations for decision-making rules. Abnormal and normal actions for the person being monitored will be detected for every detailed moment by employing a Hidden Markov Model (*HMM*).

In order to describe Hidden Markov Chains, we define *two states*: *S* = {*S*_1_,*S*_2_}. Let *S*_1_ be an abnormal state which includes “falling*,*” and let *S*_2_ be a normal state, including a set of actions, {“falling*,*” “lying*,*” “lying to sitting*,*” “sitting to standing*,*” “walking*,*” “walking to sitting*,*” “sitting*,*” and “sitting to lying”}. The process would be started in one of these two states, and proceed smoothly from one state to another. If the chain were presently in state *S*_1_, then it would proceed to the next state *S*_2_ with a specific probability. However, it should be noted that this probability will not be based on which state the chain was in before the current state.

**Figure 8:**
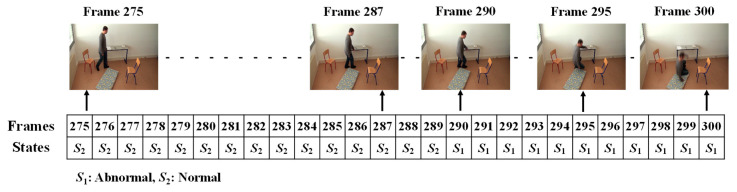
Observed sequence of the occurrences of states.

In order to form the Markov Transition matrix, we firstly observe each of the given videos by developing fixed intervals. The video sequence will become a sequence of intervals, such as *t*, *t* + *d*, *t* + 2*d*, *t* + 3*d*, …, *t* + *nd*. Here, *d* is the length of the interval to be predefined, in which only one abnormal or normal state occurs. The video sequences are manually observed to obtain the occurrence state symbol for every interval. These video sequences provide the sequences of the occurrences of the states, *S* = {*S*_1_..*S*_2_}, as illustrated in Figure 8. In this figure, S_1_ represents the abnormal state and S_2_ represents the normal state.

Then, the co-occurrence matrix *M* is constructed as shown below:

(13) $$M=\left[\begin{array}{cc} C_{11} & C_{12}\\ C_{21} & C_{22} \end{array}\right],$$

where *C*_11_ represents the number of pairs (*S*_1_,*S*_1_), *C*_12_ represents the number of pairs (*S*_1_,*S*_2_), *C*_21_ represents the number of pairs (*S*_2_,*S*_1_), and *C*_22_ represents the number of pairs (*S*_2_,*S*_2_). After that, each row of matrix *M* is summed as shown in the following:

(14) $$C_{1}=\sum \left(C_{11}+C_{12}\right),\;C_{2}=\sum \left(C_{21}+C_{22}\right),.$$

To form the Markov transition matrix, we defined the following:

(15) $$a_{11}=\frac{C_{11}}{C_{1}},\;a_{12}=\frac{C_{12}}{C_{1}},\;a_{21}=\frac{C_{21}}{C_{2}},\;a_{22}=\frac{C_{22}}{C_{2}},.$$

Then, the state transition matrix of the Markov Chain for the Hidden Markov Model (*HMM*) is obtained as shown in Equation (16):

(16) $$A=\left[\begin{array}{cc} a_{11} & a_{12}\\ a_{21} & a_{22} \end{array}\right],$$

where the elements in the first row of matrix *A* represent the probabilities for the actions following an abnormal action or fall. Similarly, the elements in the second row represent the probabilities for the actions following normal actions.

**Figure 9:**
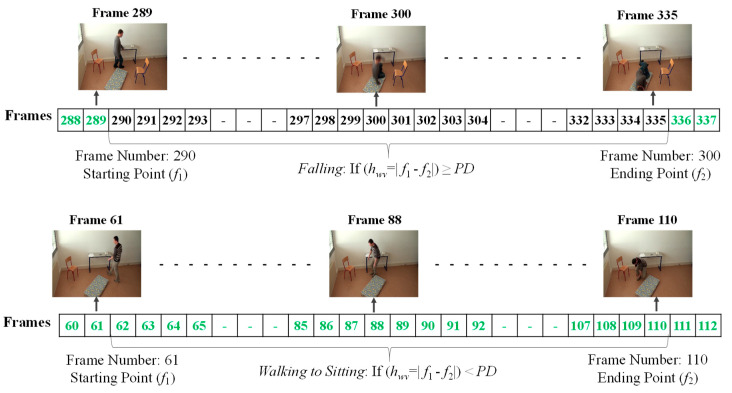
Observations in the period of abnormal and normal states. Upper part: falling; lower part: walking to sitting.

For each of the possible states, emission probabilities are calculated. We define the observable features with respect to the distance between the centroid and the corresponding virtual grounding point (*d*), the area of the object shape (*a*), and the aspect ratio (*r*). To define the observable symbols, a suitable threshold value for each of the features is selected. In doing so, we firstly calculate the features for extracting the falling period for a person, as discussed in Section 3.3. These falling periods can determine that each of the observed videos includes an abnormal or normal event. The frame index numbers through the video sequences represent that a fall can be extracted between starting point *f*_1_ and ending point *f*_2_, as illustrated in Figure 9.

In the upper part of Figure 9, the frame numbers marked in green represent the normal activities of a person, such as walking, standing, sitting, and lying. The frame numbers marked by black represent abnormal or falling events. The period of a fall might be obtained when the half width value (*h_wv_*), referring to the distance between a starting point (*f*_1_) and an ending point (*f*_2_), is greater than or equal to period detection (*PD*), as formulated in Section 3.3. Similarly, in the lower part of Figure 9, the frame numbers represent the normal activities of a person. When the person is involved in activities such as walking, walking to sitting, and sitting to standing, the analyzed half width value (*h_wv_*) is less than the detected period.

After that, it is determined whether the action is abnormal or normal by observing the six possible feature values defined below. Satisfying the criteria for this determination involves passing the selected value for threshold *F* in the consecutive frames from starting to ending points for abnormal and normal events based on the observed half width value, which determines the period of events.

*F*_1_ = {*f*_1 . ._*f*_2_} where *v_hw_* ≥ *PD*(*d*),  *F*_2_ = {*f*_1 . ._*f*_2_} where *v_hw_* < *PD*(*d*),

*F*_3_ = {*f*_1 . ._*f*_2_} where *v_hw_* ≥ *PD*(*a*),  *F*_4_ = {*f*_1 . ._*f*_2_} where *v_hw_* < *PD*(*a*),

*F*_5_ = {*f*_1 . ._*f*_2_} where *v_hw_* ≥ *PD*(*r*),  *F*_6_ = {*f*_1 . ._*f*_2_} where *v_hw_* < *PD*(*r*),

where the symbol *F* denotes that for the observed features for *HMM*, the set {*f*_1 . ._ *f*_2_} represents the sequential falling frames from starting point *f*_1_ to ending point *f*_2_. *PD*(*d*), *PD*(*a*), and *PD*(*r*) denote the period detection for a fall or an abnormal event with respect to the distance between the centroid and the corresponding *VGP*, the area of the shape, and the aspect ratio of the person, respectively, and *v_hw_* means the half width value employed to determine the period of the abnormal event.

**Figure 10:**
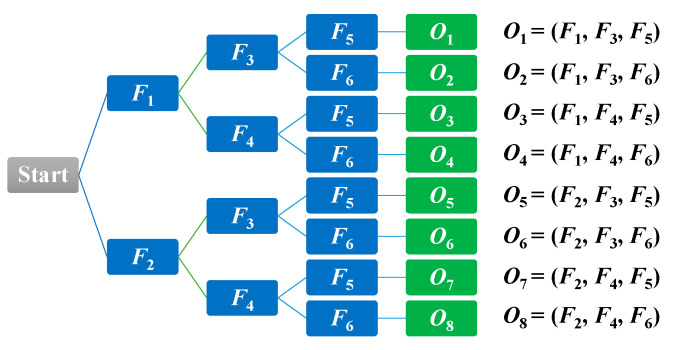
Hierarchical tree structure of independent observable symbol *O*.

After obtaining thresholds for features, calculations of emission probabilities are performed for developing the *HMM* model. The hierarchical tree structure used to form features for independent observable symbols *O* is illustrated in Figure 10.

When studying the observable symbols, we can see that *F*_1_ and *F*_2_, *F*_3_ and *F*_4_, and *F*_5_ and *F*_6_ are all disjointed. The occurrence of an observable symbol is then labeled based on the eight observable symbols *O* = {*o*_1_,*o*_2_,*o*_3_,*o*_4_,*o*_5_,*o*_6_,*o*_7_,*o*_8_} for the video sequence. The emission probability of observed symbol *o_k_* (*k* = 1,2,…,8) in state *S_j_* (*j* = 1,2) is denoted as in Equation (17).

(17) $$b_{j}\left(k\right)=\frac{\mathrm{Expected}\;\mathrm{Number}\;\mathrm{of}\;\mathrm{Occurrences}\;\mathrm{of}\;o_{k}\;\in \;\mathrm{States}\;S_{j}}{\mathrm{Expected}\;\mathrm{Number}\;\mathrm{of}\;\mathrm{Occurrences}\;\mathrm{of}\;\mathrm{States}\;S_{j}},$$

The emission probability matrix *B* is then obtained by using the following:

(18) $$B=\left[\begin{array}{cccccccc} b_{1}\left(1\right) & b_{1}\left(2\right) & b_{1}\left(3\right) & b_{1}\left(4\right) & b_{1}\left(5\right) & b_{1}\left(6\right) & b_{1}\left(7\right) & b_{1}\left(8\right)\\ b_{2}\left(1\right) & b_{2}\left(2\right) & b_{2}\left(3\right) & b_{2}\left(4\right) & b_{2}\left(5\right) & b_{2}\left(6\right) & b_{2}\left(7\right) & b_{2}\left(8\right) \end{array}\right],.$$

Then, the initial probability states are assumed to be *Prob*(*S*_1_) = 0.8 and *Prob*(*S*_2_) = 0.2. Denoting the initial probability vector as *π* gives the Hidden Markov Model (*HMM*), as shown in Equation (19):

(19) λ=(A,B,π),

where *π* is the initial probability vector.

After that, the Viterbi algorithm [27] is applied to solve the *HMM* model to detect consecutive moments in the actions. The idea is to compute the best hidden state sequence (abnormal and normal states) in the given observation sequences and in an *HMM*.

## 4. Experiments

### 4.1. The Dataset

In order to demonstrate a sub-sequential experiment, we used the publicly available Le2i fall detection dataset [28], which simulates the activities of the elderly in a home environment, as well as in an office. This dataset was developed for detecting falls using artificial vision, which can be useful in helping the elderly. These simulated videos illustrate the difficulties in creating realistic video sequences that include variable illumination, occlusions, and cluttered or textured backgrounds. The actors performed various normal daily activities and falls, in an attempt to simulate elderly behavior. In addition, the actors did not perform acts that would be unnatural for the elderly, such as running or walking quickly. The video sequences were captured using a single RGB camera, in which the frame rate was 25 frames per second, and the resolution was 320 × 240 pixels. From this dataset, 20 videos taken in an office were selected, representing normal daily activities and falls as performed by four different actors.

**Figure 11:**
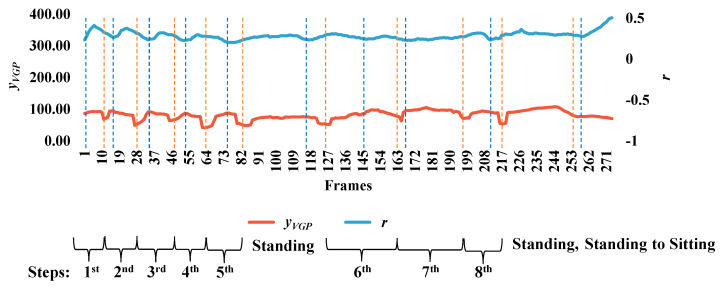
Two extracted features—*y_VGP_* (*y* coordinate of the virtual grounding point (*VGP*)) and aspect ratio (*r*)—which can provide information on the number of steps for a person.

**Figure 12:**
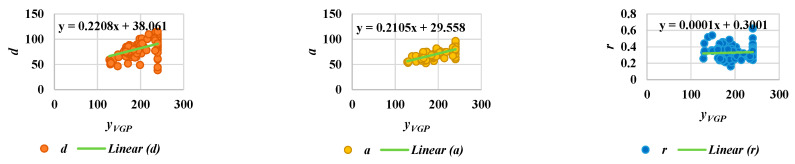
Analysis of camera calibration for all scenes.

Acquiring useful qualitative data for surveillance systems is very important in measuring distances, positions, velocities, and directions for the objects in the scenes, and is a necessary consideration in developing reliable systems [29]. As an important contribution of the current work, we have developed guidelines for setting up and calibrating cameras. First of all, it is essential to try to confirm whether or not the camera height and angle are approximately the same for all videos. We can derive an approximate linear equation for camera calibration. To do so, two extracted features for the object are employed, namely, the aspect ratio (*r*), and *y_VGP_*, corresponding to the *y* coordinate. These two features can provide information on steps in the walking pattern for a person, as described in Figure 11. In Figure 11, the person exhibits the same posture corresponding to *y_VGP_* and *r*, as illustrated by the blue and orange dashed lines. The interval of the step can be obtained from the place between where the same postures occur. Then, we can observe the index numbers for frames and the corresponding values for the point distance (*d*), area of the object shape (*a*), and aspect ratio (*r*)*,* with respect to starting and ending frames in the step intervals. In order to formulate a linear equation for camera calibration, we assume three kinds of points: (*y_VGP_*,*d*), (*y_VGP_*,*a*), and (*y_VGP_*,*r*), as shown in Figure 12. Then, by observing the testing points, Figure 12 shows that no significant differences exist between the variation within a scene and the variation between all scenes.

### 4.2. Experimental Results

**Figure 13:**
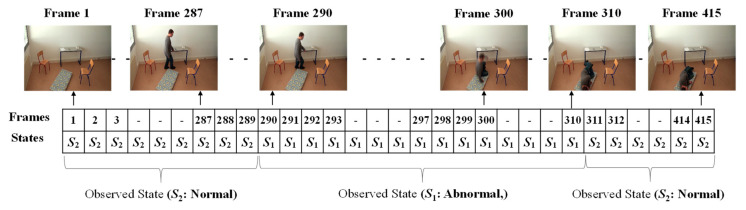
Observations of the occurrences of states for Mr. ONE (Video 1).

The given video was firstly analyzed by setting a fixed interval. Here, we made the interval every frame in which only one state occurs (*S*_1_: abnormal or *S*_2_: normal). Manual observations of the video sequences were performed to provide the occurrence state symbol for every interval or frame. Such observations for a video sequence used in these experiments are illustrated in Figure 13. For effectively demonstrating the consequences of the sub-sequential experimental results, here, we denote the name of the person in the video sequence as “Mr. ONE.”

After that, as discussed in Equation (16), the state transition matrix of the Markov Chain for the *HMM* was obtained by applying Equations (13)–(15).

After making observations of the occurrence state symbols (*S*_1_,*S*_2_), the co-occurrence matrix was constructed by applying Equation (13), as shown below.

$$M=\left[\begin{array}{cc} 900 & 11550\\ 11550 & 148225 \end{array}\right]$$

Then, the following values were obtained using Equations (14) and (15):

$$a_{11}=c_{11}/c_{1}=0.07229,\;a_{12}=c_{12}/c_{1}=0.92771,\;a_{21}=c_{21}/c_{2}=0.07229,\;a_{22}=c_{22}/c_{2}=0.92771.$$

After that, we obtained the transition probability matrix *A*(*a_ij_*), which represents the probability of proceeding from state *S_i_* to state *Sj*, by computing Equation (16).

$$A=\left[\begin{array}{cc} 0.07229 & 0.92771\\ 0.07229 & 0.92771 \end{array}\right]$$

**Figure 14:**
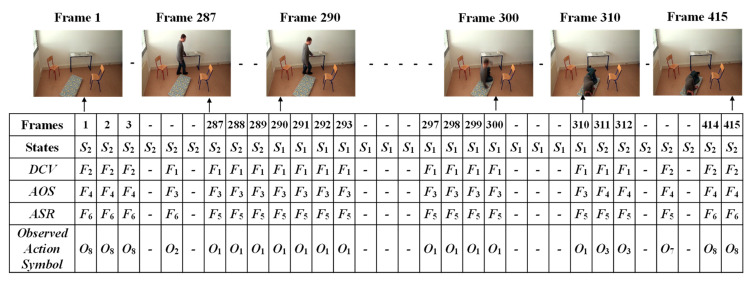
Observations for independent action symbol *O* for Mr. ONE (Video 1).

By employing Equations (17) and (18), the independent observable symbols *O* were obtained as shown in Figure 14. After that, the occurrence of an observable symbol was identified using one of the labels for the eight observable symbols {*o*_1_,*o*_2_,*o*_3_,*o*_4_,*o*_5_,*o*_6_,*o*_7_,*o*_8_} for the video sequence. The emission probability matrix *B* was then obtained as shown below:

$$B=\left[\begin{array}{cccccccc} 0.75 & 0 & 0.25 & 0 & 0 & 0 & 0 & 0\\ 0.020672 & 0.062016 & 0.015504 & 0 & 0 & 0.069767 & 0.054264 & 0.777778 \end{array}\right].$$

Matrix *B* represents a sequence of observation likelihoods in which each element expresses the probability of an observation *o_k_* (*k* = 1,2,…,8) being generated from a state *S_j_* (*j* = 1,2).

**Figure 15:**
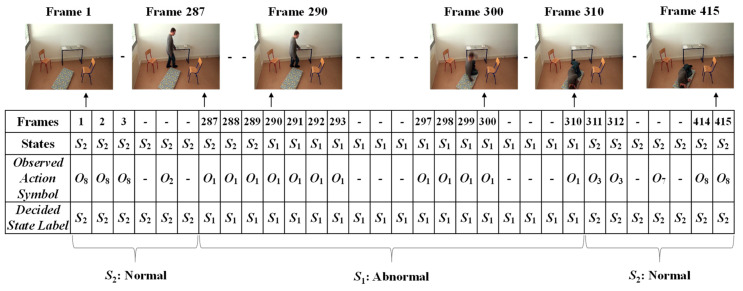
Results for deciding on the sequential abnormal and normal states for Mr. ONE (Video 1).

**Figure 16:**
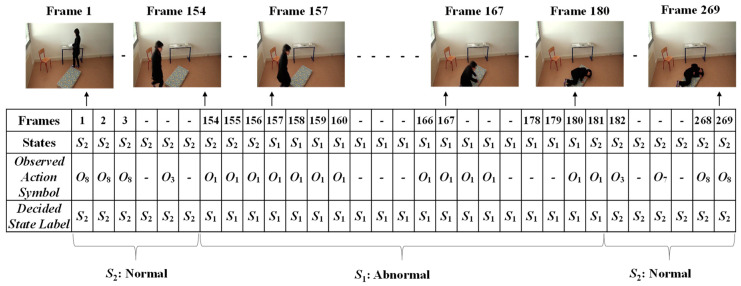
Results for deciding on the sequential abnormal and normal states for Ms. TWO (Video 1).

**Figure 17:**
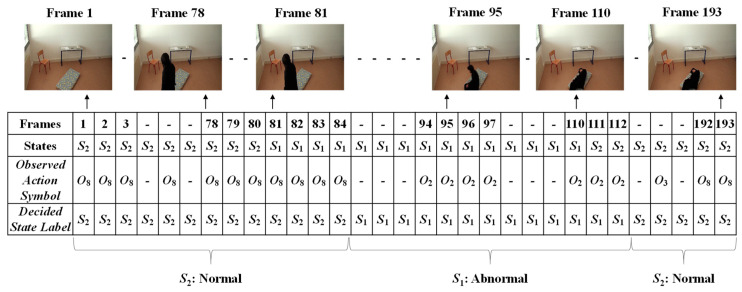
Results for deciding on the sequential abnormal and normal states for Ms. TWO (Video 2).

**Figure 18:**
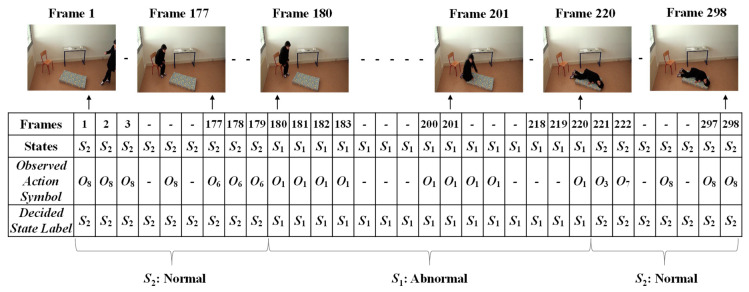
Results for deciding on the sequential abnormal and normal states for Ms. TWO (Video 3).

Finally, the Viterbi algorithm [27] was employed for solving the *HMM* model to recognize sequential abnormal and normal states for “Mr. ONE.” The input parameters for implementing the Viterbi algorithms were summarized as two states—abnormal and normal—including eight observable action symbols; {“falling*,*” “lying*,*” “lying to sitting*,*” “sitting to standing*,*” “walking*,*” “walking to sitting,” “sitting*,*” and “sitting to lying”}, a state transition probability matrix (*A*), a sequence of observation likelihood or emission probability matrix (*B*), and an initial probability distribution over states. The final outcome results for “Mr. ONE” are illustrated in Figure 15. Additional experimental results for sequential abnormal and normal states in each of the video sequences simulated by Ms. TWO are demonstrated in Figure 16, Figure 17 and Figure 18, respectively.

### 4.3. Performance Evaluation of the Proposed Approach

In order to evaluate the performance of our proposed system, we firstly used the detection process for falling and normal events using three-fold cross-validation, which presents the swapped variables for learning and testing used in our previous work [13]. The given videos were classified as either falling or non-falling videos. Our proposed period detection (*PD*) achieved a precision of 93.33% and a recall of 93.33%. In the current work, we enhanced our detection system “from video to consecutive video sequences” by utilizing the analyzed features working for the *PD*. The features were used as inputs to calculate the probability for *HMM*. There are four possible outcomes in discriminating abnormal and normal states for the consecutive states of a person, and definitions and relative symbols are described in the following:

- Detected Abnormal State (*As*_1_): A video frame represents an abnormal state, and is correctly classified as “Positive Abnormal”;
- Undetected Abnormal State (*As*_2_): A video frame represents an abnormal state, and is incorrectly classified as “Negative Normal”;
- Normal State (*Ns*_1_): A video frame does not represent an abnormal state, and is correctly classified as “Negative Normal”;
- Mis-detected Normal State (*Ns*_2_): A video frame does not represent an abnormal state, and is incorrectly classified as “Positive Abnormal”.

The following Equations (20)–(24) describe as a performance evaluation the calculated precision, recall, accuracy, specificity rate, and negative predictive value, respectively. In evaluating each of the given videos, an “undecided class” is nominated in order to save the unnecessary failed states. In other words, where we consider the risk to be low for a given video, we ignore it when a normal case is misclassified as abnormal.

(20) $$\mathrm{Pr}ecision=\frac{A_{s1}}{\left(A_{s1}+N_{s2}\right)}*100,$$

(21) Recall=As1(As1+As2)*100,

(22) $$Accuracy=\frac{\left(A_{s1}+N_{s1}\right)}{\left(A_{s1}+N_{s1}\right)}*100,$$

(23) $$SpecificityRate=\frac{N_{s1}}{\left(N_{s1}+N_{s2}\right)}*100,$$

(24) $$NPV=\frac{N_{s1}}{\left(N_{s1}+A_{s2}\right)}*100,$$

**Figure 19:**
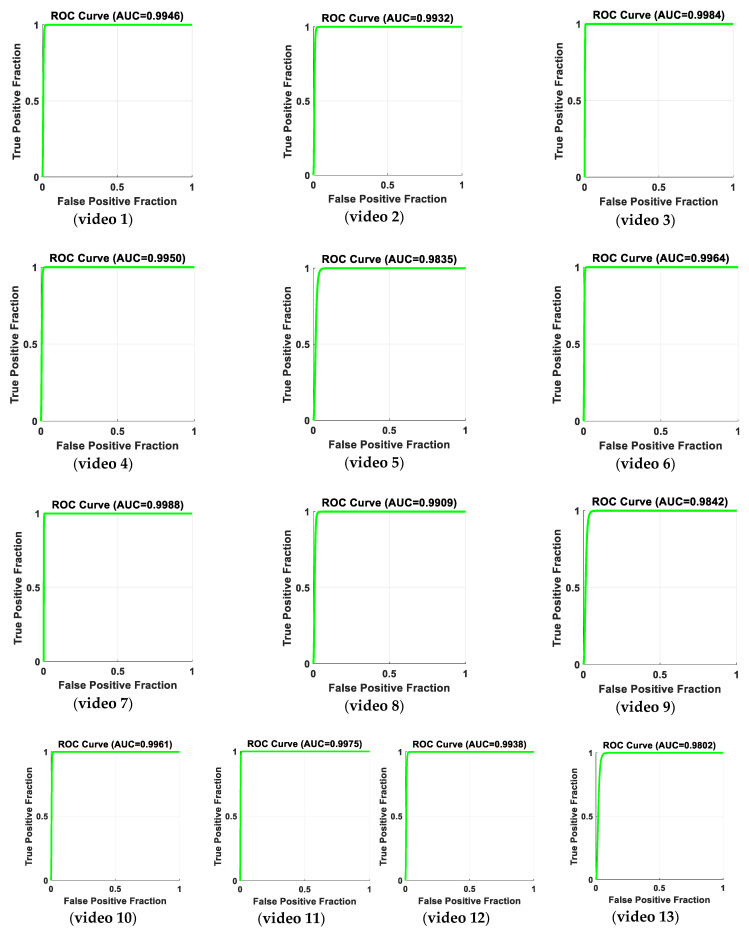
Receiver operating characteristic (*ROC*) curve analysis for the results of the true positive fraction (*A_s_*_1_/(*A_s_*_1_ + *A_s_*_2_)), and false positive fraction (*N_s_*_2_/(*N_s_*_2_ + *N_s_*_1_)), for 13 videos including abnormal and normal events. The results of *AUC* (area under *ROC* curve) with respect to the true positive fraction and false positive fraction show that our proposed method is quite capable of distinguishing between the abnormal and normal states.

Table 1 provides the precision, recall, accuracy, specificity rate, and negative predicted value (*NPV*) for 13 videos, including both abnormal and normal states. The remaining seven videos that include a normal state provide a precision of 100% and a recall of 100%. Figure 19 shows receiver operating characteristic (*ROC*) curve analyses for the results of true positive and false positive fractions, for the 13 videos that include abnormal events. The results of *AUC* (area under *ROC* curve) show that our proposed method is quite capable of distinguishing between the classes. A comparison of the performance of the proposed system for each video and related existing methods is shown in Table 2.

Our proposed system was implemented in MATLAB 2019a on an academic license. A series of experiments were performed on Microsoft Windows 10 Pro with an Intel (R) Core (TM) i7-4790 CPU@3.60 GHz and 8GB RAM. The overall computation time in this system is 0.93 s per frame. In order to achieve real-time monitoring, we expect that the GPU implementation could be further fine-tuned to our system.

### 4.4. Comparative Studies of the Merits and Demerits of Our Proposed System

Kishanprasad presented a fall-detection system [16] providing a precision of 94% and a recall of 95%, as tested in an office environment from the Le2i dataset. The merits of this system were described as robust, fast, and computationally efficient. The system could be enhanced using deep learning models in future work. Demerits of the system were not discussed in detail. However, our proposed system achieved higher precision and recall rates, as shown in Table 2.

Suad [18] proposed a fall detection approach using threshold-based methods and a neural network. The system achieved a high detection rate in monitoring a single person in a home environment. However, their proposed system can only detect events in high-intensity light. They mentioned ways to enhance their proposed system, such as using it to detect multiple people and installing multiple cameras to make sure that the object is visible in at least one camera view. Moreover, they have mentioned adopting enhanced background subtraction for better object segmentation. Other improvements can be achieved by detecting additional features using sensors such as an accelerometer. Capturing 3D information using a depth camera could also improve the detection rate. Using simple algorithms, we achieved an accuracy similar to that of Suad’s proposed system, not attaining the accuracy of our proposed system.

Adrian [23] presented a vision-based fall detection system using *CNN* architecture. As one drawback of the system, using optical flow images involves a heavy computational burden in generating consecutive sequences. Furthermore, the system is not robust when lighting changes. Therefore, a more reliable network could be modeled to gain a better representation of hierarchical motion from the images. In addition, more research involving multiple people in falling events would be useful before implementation in public areas. Compared to this method, our proposed system gives more accurate rates.

Minjie’s work [24] investigated an approach for predicting falling events in advance using monocular videos. The system has credibility as it provided excellent performance results using a complex system architecture. However, they described two failures due to the non-detection of keypoints, which occurred when the person’s upper body was not visible from the perspective of the camera. Moreover, falls cannot be predicted in advance if the precursor of the fall is not detected. In comparison with our proposed system, we achieved a higher precision, recall, and overall accuracy, as described in Table 2.

In this paper, we have proposed a system for detecting normal, abnormal, and falling states using video sequences for people being monitored. In detecting normal states, our system outperformed other approaches, with an accuracy of 99.8%, using experimental videos containing non-abnormal events. In addition, our proposed system proved to be reliable and effective, with a precision of 99.05% and recall of 98.37%, as confirmed using 20 video sequences that contained abnormal and normal events. Though the system was adapted to a real-world environment, the scope of research was limited to detecting a single person. The computation time for the object detection module is slow in a real-time monitoring system. Using multiple cameras to view the human body from different perspectives would improve feature extraction. Currently, the system is only effective during the day, and should be improved for night-time use in the real world of elderly care. Moreover, the system functionality should be expanded by exploring feature analyses such as pre-fall states, dangerous post-fall situations, and abnormal gaits (walking patterns). We expect that smart image processing technologies have great potential for improving the quality of life of the targeted population.

## 5. Discussion and Conclusions

The research discussed in this paper is concentrated on applied statistical analysis for developing an advanced image-processing technology in vision-based elderly care monitoring systems, facilitating health care for elderly people living independently. In brief, our proposed approach involves the following. Background subtraction is first used to detect moving and motionless objects. Simple and effective feature extraction is then performed using the virtual grounding point concept (*VGP*), as well as the area of the shape and the aspect ratio of the detected object. In addition, these three features are observed in detailed considerations of the moving average and modified difference calculations. Careful observations of the critical features of the local maximum or local minimum are made to predict the high possibility of an abnormal state for the point distance of the *VGP*, area, and aspect ratio. Moreover, the optimal threshold values are exploited by calculating the mid value or period detection for the probability distribution of the Hidden Markov Model. Finally, the consecutive abnormal and normal states are differentiated using the Viterbi algorithm. Contributing modules include an analysis of the state with an extensive observable area of shape features and making decision rules using the Hidden Markov model. Moreover, a simple analysis of camera calibration is performed using our observed *VGP* and its inclusive features. Experimental results indicate that our proposed system is reliable and effective for detection. In order to confirm the validity of our proposed methods, we have drawn comparisons with existing methods described in the literature. Specifically, comparisons were made between our approach and three similar methods: (1) threshold-based fall detection; (2) neural network algorithms [16,18]; and (3) the *CNN* approach [23,24]. Compared to these methods, our proposed method achieved a higher performance, as confirmed using the public dataset of videos taken in simulated environments that closely approximate real life. This paper focuses on detecting falls, which are common among elderly people. In future research, we will improve our analysis by applying it to behavioral features, such as timing, posture, and gait. Using the results of surveys, we will also take into consideration behavioral differences between healthy young adults and the elderly. Though human behavior is complex and inconsistent between different persons, situations, and environments, our system can improve the interpretation of human behavior through an analysis of its visual manifestations in different situations. However, further testing using large amounts of data is required for system optimization. Furthermore, we expect that our proposed system can be improved by applying techniques related to modern deep learning. In fact, future experiments involving simulations of abnormal events should prove a fruitful area for applying such techniques.

## Figures and Tables

**Table 1:** Performance evaluation for 13 fall video sequences.

Videos	Precision (%)	Recall (%)	Accuracy (%)	Specificity (%)	NPV (%)
1	100	100	100	96.88	96.88
2	100	100	100	96.07	96.09
3	100	86.66	98.32	99.04	97.17
4	100	100	100	97.07	97.06
5	100	100	100	90.40	90.40
6	82.98	97.50	99.76	97.89	97.63
7	98.04	100	100	99.36	99.36
8	100	100	100	94.70	94.70
9	100	83.33	97.41	90.80	99.09
10	100	100	100	97.72	97.72
11	100	100	100	98.54	98.54
12	100	100	100	96.38	96.38
13	100	100	100	88.48	88.48

**Table 2:** Performance of our system compared with existing methods using the same dataset.

Methods	Precision (%)	Recall (%)	Accuracy (%)
Kishanprasad [16]	94	95	—^1^
Suad [18]	100	95.27	99.82
Adrián [23]	—^1^	99.00	97.00
Minjie [24]	90.8	98.3	97.8
Ours	99.05	98.37	99.8

Note: —^1^ means the provided data is not available.
